# Genetic Mapping Identifies Stable QTL and Candidate Genes Regulating Internode Proportion for Maize Plant Architecture Improvement

**DOI:** 10.3390/genes17020141

**Published:** 2026-01-27

**Authors:** Xueying Li, Hao Zhang, Keying Wan, Xiaoqian Qiu, Qiankun Xie, Geming Guo, Yuehua Zhao, Zibo Ding, Xiaoyang Chen, Hongyu Chen, Huiling Xie, Jihua Tang, Xuehai Zhang, Dong Ding

**Affiliations:** 1 State Key Laboratory of High-Efficiency Production of Wheat-Maize Double Cropping, College of Agronomy, Henan Agricultural University, Zhengzhou 450046, Chinatangjihua1@163.com (J.T.); 2 The Shennong Laboratory, Zhengzhou 450046, China

**Keywords:** maize, internode proportion, plant architecture, genome-wide association studies, quantitative trait loci

## Abstract

Background: Ideal plant architecture is central to high-yield maize breeding. The proportional length of internodes above the ear plays a crucial role in determining plant architecture. Methods: In this study, we used an association panel comprising 288 maize inbred lines and performed genome-wide association studies (GWASs) with 1.25 M high-density single nucleotide polymorphism (SNP) markers under a Q + K mixed linear model. Results: A total of 821 significant SNPs associated with plant height (PH), height above ear (HAE), and internode-related traits were detected, which were further classified into 417 quantitative trait loci (QTL). Among these, 128 significant SNPs and 44 QTL were identified for the U1/HAE trait, and 37 significant SNPs and 27 QTL for the U1/PH trait. Four stable QTL (*qU1*–*qU4*) were identified through colocalization analysis. Two candidate genes, *Zm00001d013222* (involved in gibberellin signaling) and *Zm00001d021304* (involved in cell wall metabolism), were further supported by haplotype analysis. For the former gene, U1/PH values in Hap1 and Hap3 were significantly lower than those in Hap2 (*p* < 0.01). For the latter gene, Hap2 exhibited a significantly higher U1/HAE value compared to Hap1 (*p* < 0.001). Conclusions: These findings provide new genetic insights into the regulation of maize internode proportion and plant architecture, offering potential targets for molecular breeding.

## 1. Introduction

Plant height (PH) and internode length (IL) are key determinants of ideal plant architecture and are closely associated with crop yield and plant adaptability. In maize, optimizing plant architecture has become a major focus in breeding programs to enhance productivity and resilience under varying environmental conditions [1]. As one of the world’s most important cereal crops, maize is frequently subjected to abiotic stresses that can compromise structural stability and ultimately limit yield [2].

Genetic studies have identified several genes that regulate maize internode elongation and plant stature. For example, the dwarf gene *Brachytic2* (*br2*) shortens lower internodes and alters stem morphology [3], while its allele *qpa1* reduces PH and ear height (EH) and increases stem thickness [4]. The *gif1* gene promotes cell proliferation in stems, and its mutant exhibits shortened internodes [5]. Similarly, transcription factors such as *BLH12* and *BLH14* interact with *KNOTTED1* to maintain meristem activity and ensure normal stem elongation [6]. These findings underscore the genetic complexity underlying internode development and its importance in shaping plant architecture.

Internode elongation is coordinately regulated by a network of hormone signaling pathways, including auxin, gibberellin (GA), brassinosteroid (BR), ethylene, jasmonic acid (JA), and strigolactone (SL) [7]. For instance, the *GRAS42* gene modulates internode development through BR-mediated signaling and cell wall biosynthesis [8]. Mutants of *GRAS42* exhibit a dwarf phenotype, with plant height reduced to approximately 60% of the wild type due to shortened internodes [9]. This effect is further enhanced in the *nana plant1-1* and *gras42-mu1021149* double mutant, underscoring the gene’s role in internode regulation [9]. Similarly, disrupted GA signaling inhibits internode elongation and reduces plant height [10], while the jasmonate analog Coronavirin suppresses maize internode elongation by downregulating the cell wall-associated gene *ZmXTH1* [11]. Ethylene also contributes to this process, as evidenced by the *ZmACS7* gene, which influences the elongation of both internode and auricle cells [12]. Additionally, strigolactone biosynthesis is critical for normal stem development; mutations in *ZmCCD8*, which encodes a carotenoid cleavage dioxygenase, impair SL production and lead to reduced stem diameter, compromised internode elongation, and overall plant dwarfism [13]. Collectively, these findings illustrate the sophisticated hormonal crosstalk that integrates multiple pathways to coordinately control stem growth and architecture.

Despite advances in understanding PH genetics, the genetic basis of specific internode proportions—particularly those above the ear—remains underexplored. Traditional studies often focus on overall PH or ear height, yet the proportional length of individual internodes may offer more precise targets for architectural improvement. For example, the first internode above the ear (U1) has been noted for its structural importance and susceptibility to environmental stress [14,15]. Studies have demonstrated that knockout of the *ZmD1* gene suppresses longitudinal elongation while promoting transverse expansion of internode cells, leading to reduced plant height and shortened internodes [16]. The *ZmTE1* gene plays a crucial role in maintaining internode meristem formation and cell division and regulates internode elongation by promoting cell extension [17]. Notably, in maize breeding research, three quantitative trait loci (QTL) associated with internode length have been identified, from which 303 related genes have been annotated [18]. These genes influence internode elongation primarily by mediating phytohormone signaling, modulating receptor activity, and regulating carbon metabolism pathways. Among them, *ZmIL1*, *ZmIL2*, and *ZmIL3* have been highlighted as key candidate regulators of internode length [18].

In this study, we employed a GWAS approach using a diverse association panel of 288 inbred lines genotyped with 1.25 M SNPs to dissect the genetic architecture of internode proportion traits, with a particular focus on U1/HAE and U1/PH. Our objectives were to (1) characterize phenotypic variation in internode-related traits across two environments; (2) identify significant SNPs and stable QTL associated with internode proportions; and (3) pinpoint candidate genes through integrated bioinformatic and haplotype analyses. This work provides new insights into the genetic regulation of maize plant architecture and offers candidate loci for molecular breeding.

## 2. Materials and Methods

### 2.1. Plant Materials and Experimental Design

The association mapping panel consisted of 288 maize inbred lines, including 131 tropical/subtropical and 157 temperate accessions. In 2024, the panel was planted at two field locations: Yuanyang Modern Agricultural Science and Technology Park (35° N, 113° E; designated as 24YY; average altitude: 74 m; mean annual temperature: 14.3 °C; mean annual precipitation: 550 mm; soil organic matter: 10.6 g·kg^−1^; total nitrogen: 1.1 g·kg^−1^) and Zhangye, Gansu (39° N, 100° E; designated as 24GS; 39° N, 100° E; designated 24GS; average altitude: 1400–1500 m; mean annual temperature: 8.4 °C; mean annual precipitation: 197.4 mm; soil organic matter: 18.0 g·kg^−1^; total nitrogen: 1.1 g·kg^−1^). Each inbred line was replicated twice per location in a full randomized block design, with a row length of 3.0 m, plant spacing of 0.25 m, and row spacing of 0.67 m. Standard field management practices were followed throughout the growing season.

### 2.2. Phenotypic Data Collection

At physiological maturity, five representative plants per row were selected for phenotyping. Plant height (PH) and ear height (EH) were measured in the field for each plant. Subsequently, whole stalks were harvested and transferred to the laboratory for detailed internode measurements. Internode traits included the length of the ear-position internode (designated Zero), the lengths of the five internodes above the ear (U1–U5), and the five below the ear (B1–B5). (Supplementary Figure S1).

### 2.3. Phenotypic Data Analysis

For each trait, the mean value per inbred line was first calculated from five plants within each environment. All general statistical analyses in this study were conducted using the spreadsheet program within the WPS Office 12.1.0 software suite (Microsoft Excel-compatible). For maize trait measurements, descriptive statistics were calculated as follows: the mean was computed using the AVERAGE function, skewness and kurtosis were determined with the SKEW and KURT functions, respectively, and the sample standard deviation was derived using the STDEV.S function. The coefficient of variation (CV) was calculated as:

$$CV(\%)=\frac{Standard\;Deviation}{Mean}\;\times \;100\%$$

To assess environmental effects, Pearson correlation between traits across the two locations was evaluated using the cor() function in R (v4.3.1). Best linear unbiased predictions (BLUPs) for each trait across the two environments (24GS and 24YY) were then estimated using the lme4 package, with genotype as a random effect and environment as a fixed effect, the broad-sense heritability (*h*^2^) for each trait was calculated using the following formula:

$$h^{2}\;=\;\frac{V_{G}}{V_{G}\;+\;V_{\mathrm{GE}}/n\;+\;V_{e}/(\mathrm{nr})}$$

where $V_{G}$ is the genotypic variance, $V_{\mathrm{GE}}$ is the genotype × environment interaction variance, $V_{e}$ is the residual error variance, *n* is the number of environments, and *r* is the number of replications. Variance components were estimated using analyzed by analysis of variance (ANOVA) using the aov() function in R (v4.3.1). Both the environment-specific line means and the across-environment BLUP were used as phenotypic inputs in the subsequent genome-wide association analysis. Frequency distributions of phenotypic data were visualized with GraphPad Prism 10.1.2.

### 2.4. Genome-Wide Association Study (GWAS)

Genotype data for the association panel, consisting of approximately 1.25 million (1.25M) single nucleotide polymorphism (SNP) markers aligned to the B73 RefGen_v4 reference genome, along with corresponding population structure information, were obtained from the publicly available accessible dataset provided by Prof. Jianbing Yan of Huazhong Agricultural University. The dataset is available for download at http://www.maizego.org/Resources.html (accessed on 20 January 2026) [19]. After rigorous quality control of each dataset, genotypes from four genotyping platforms (600K, 50K, RNA-seq, and GBS) were merged. Conflicting locus calls were resolved by prioritizing platforms in the order 600K > 50K > RNA-seq > GBS. Genotype imputation was performed using Beagle v4.0 [20]. To optimize imputation parameters and validate accuracy, chromosome 10 markers were used as a reference set [21]. A total of 15,000 known genotypes (approximately 3% of chromosome 10 loci, with three individuals per locus) were randomly masked as missing, and imputation reliability was assessed by comparing the masked true genotypes with the imputed values. Testing multiple parameter combinations identified the optimal settings: window = 50,000, overlap = 5000, ibd = true. Two imputation strategies were compared: the “remove-then-impute” strategy (filtering SNPs with >90% missing rate before imputation) achieved an average accuracy of 96.93%, whereas the “impute-then-remove” strategy (filtering after imputation) yielded a lower accuracy of 95.89% [21]. Accordingly, the former strategy was adopted for final analyses [21]. An integrated genetic map comprising 2.65 million loci was constructed for 540 individuals; of these, 1.25 million loci with minor allele frequency (MAF) ≥ 5% were retained for downstream studies [21]. The final merged HapMap-format genotype dataset is available at www.maizego.org/Resources (accessed on 20 January 2026). GWASs were performed in Tassel 5.2.81 using the Q + K mixed linear model to correct for population structure and kinship. The significance threshold was set at *p* ≤ 1.0 × 10^−5^ [18]. Result visualization—including Manhattan plots, quantile–quantile (Q-Q) plots, and composite figures—was conducted in RStudio (2025.9.2.0) with the CMplot, ggplot2, ggridges, and corrplot packages.

### 2.5. Candidate Gene Identification

For each significantly associated SNP, a 100 kb window (50 kb upstream and downstream) was defined as the QTL interval, corresponding to the average linkage disequilibrium decay distance (~50 kb) in this population [22]. Gene annotations within these regions were retrieved from the MaizeGDB database (B73 RefGen_V4, https://maizegdb.org/, accessed on 20 January 2026). Functional descriptions and expression profiles of candidate genes were examined, and expression heatmaps were generated using the Omicshare online platform (https://www.omicshare.com/tools/Home/Task, accessed on 20 January 2026).

### 2.6. Haplotype Analysis

Haplotype analysis was carried out with Tassel 5.2.81. For candidate genes, gene-based association analysis was performed by extracting gene sequences from the association panel and subsequently evaluating their statistical associations with the target phenotypic traits. Significant SNPs were used to construct haplotypes. Haplotypes represented by fewer than 10 accessions were excluded from further analysis. Phenotypic differences among haplotypes were assessed for statistical significance using a two-tailed *t*-test.

## 3. Result

### 3.1. Phenotypic Variation in Internode-Related Traits

A total of 28 internode-related traits were investigated in this study, including plant height (PH), ear height (EH), height above ear (HAE), lengths of internodes above the ear (U1–U5), lengths of internodes below the ear (B1–B5), the ear-position internode (Zero), the average length of the five internodes above the ear (U_AVE), and a series of proportional traits: U1-U5 to HAE, B1-B5 to EH, Zero to HAE, U_AVE to HAE, and U1 to PH. All traits exhibited considerable phenotypic variation across the association panel. Coefficient of variation (CV) values ranged from 0.02 to 0.28 under the two environments and their best linear unbiased predictions (BLUPs) (Table 1). Skewness and kurtosis values for each trait were generally between −1 and 1, indicating that the phenotypic distributions were approximately normal (Supplementary Figure S2) and consistent with quantitative inheritance controlled by multiple genes. Heritability was estimated for all traits under two environments, and the results showed that the heritability of different traits exhibited significant high–low differentiation characteristics between the two environments. These results confirm that the panel captures broad genetic diversity suitable for genome-wide association mapping of internode architecture.

Notably, within the same environment, internode lengths (ILs) at different positions above the ear were strongly positively correlated (Supplementary Figure S3A–C), suggesting shared genetic regulation or linkage among underlying loci. In contrast, correlations for the same IL trait across different environments were markedly lower (Supplementary Figure S3D–F), highlighting the environmental sensitivity of upper-ear internodes and the dominant role of environment in shaping their phenotypic expression.

To further evaluate the architectural role of internodes above the ear, we examined the proportional contributions of individual internodes. The ratio of the first internode length (U1) to both height above ear (HAE) and plant height (PH) was consistently and significantly higher than that of the other four internodes across environments and BLUPs (Supplementary Figure S4). This stable, dominant proportional role suggests that U1 is a structural cornerstone of the upper-plant profile and likely exerts a major influence on overall above-ear morphology. Given its pronounced and consistent phenotypic contribution [14,15], we focused subsequent genetic analyses on the traits U1/HAE and U1/PH.

### 3.2. Genome-Wide Association Analysis of Internode-Related Traits

**Figure 1:**
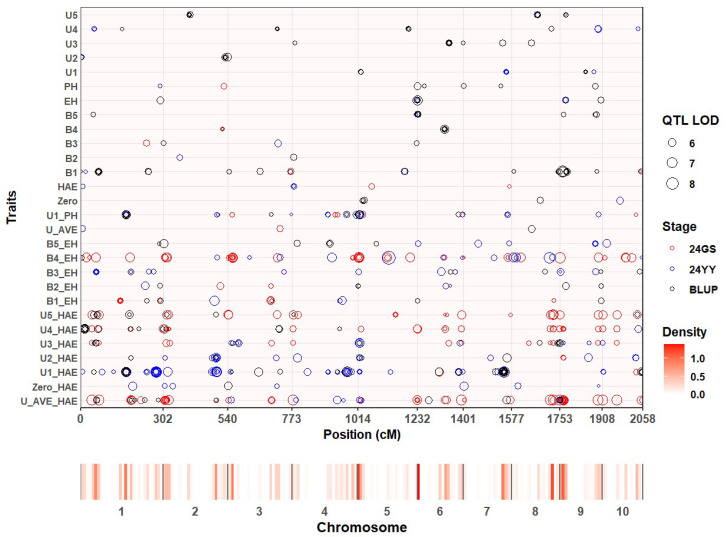
Chromosomal distribution of significant QTL associated with IL-related traits.

We performed genome-wide association studies (GWASs) on maize internode length (IL) and internode ratio traits using approximately 1.25 million SNP markers and the Q + K mixed linear model. Analyses were conducted separately for each of the two environments (24GS and 24YY) and for across-environment best linear unbiased predictions (BLUPs) (Supplementary Figures S5–S8; Supplementary Table S1). After accounting for population structure and kinship, 821 SNPs exceeded the significance threshold of −log_10_(*p*) > 5.0 (*p* < 1 × 10^−5^). These SNPs were grouped into 417 non-redundant quantitative trait loci (QTL) based on a 50 kb linkage-disequilibrium (LD) window. QTL were distributed across all ten maize chromosomes, with notable enrichment on chromosomes 1, 2, 5, and 7 (Figure 1). Among all the identified QTL, 13 stable QTL were screened through colocalization analysis across at least two environments, and these QTL intervals contained a total of 60 internode-related candidate genes (Supplementary Table S2). These associated regions provide a foundation for subsequent candidate-gene identification related to internode proportion.

**Figure 2:**
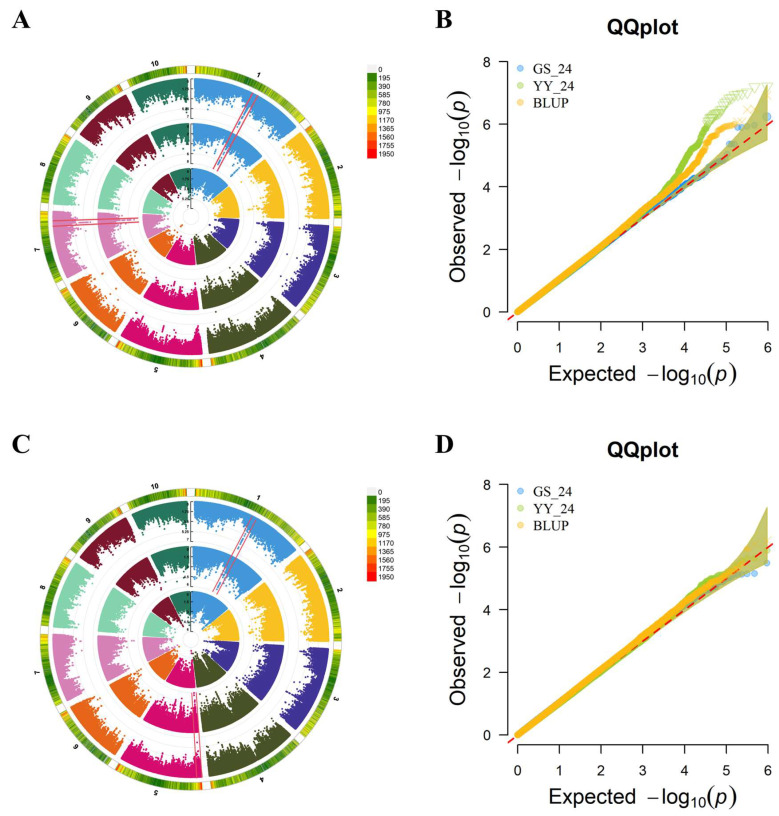
Circular manhattan plots and Q-Q plots for GWAS of U1/HAE (**A**,**B**) and U1/PH (**C**,**D**). Notes: circular manhattan plots (**A**,**C**) present genome-wide association signals across chromosomes, where the outermost ring indicates SNP density with a green-to-red gradient representing increasing density, and innermost to middle rings show association results for the 24GS, 24YY and BLUP datasets. Q-Q plots (**B**,**D**) contrast observed versus expected −log_10_(p) values for the 24GS, 24YY and BLUP datasets; the upward deviation from the diagonal confirms the presence of true genetic associations and the robustness of detected QTL across environments.

To further dissect the genetic basis of the first-internode proportion in shaping above-ear architecture, we focused GWASs on the traits U1/HAE and U1/PH. For U1/HAE, 128 significant SNPs were detected across environments and BLUPs (−log_10_(*p*) > 5.0). Based on the population LD decay distance (~50 kb), these SNPs defined 44 QTL, distributed on chromosomes 1, 2, 3, 4, 5, 6, 7, and 10. Integration of signals from the 24YY environment and the BLUP dataset revealed two consistently detected, stable QTL: *qU1* (chr1: 165.32–165.52 Mb) and *qU2* (chr7: 148.32–148.51 Mb) (Figure 2A,B). For U1/PH, 37 significant SNPs were identified, corresponding to 27 QTL located on all chromosomes except chromosome 8. Through combined analysis of the 24YY environment and BLUP data, two stable QTL were consistently mapped: *qU3* (chr1: 165.38–165.52 Mb) and *qU4* (chr5: 6.71–6.81 Mb) (Figure 2C,D). The repeated detection of these QTL across independent datasets indicates that they represent genomic regions with stable genetic effects on internode proportion.

### 3.3. Analysis of Candidate Genes for U1/HAE and U1/PH Traits

**Figure 3:**
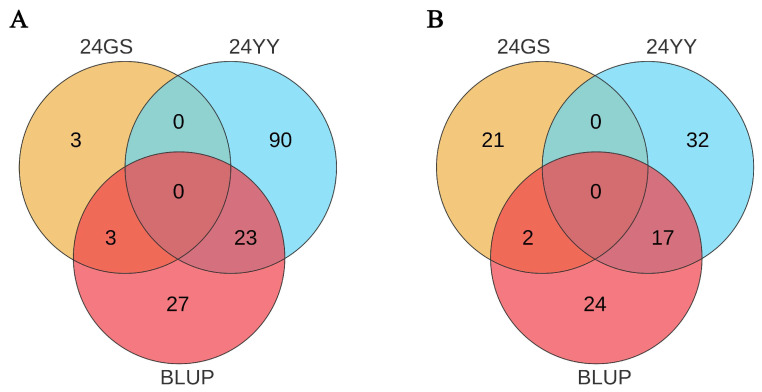
Overlap of candidate genes identified for U1/HAE and U1/PH across two environments and BLUP values. Notes: Venn diagrams illustrate the number of candidate genes located within significant loci that were detected in the two individual environments (24GS and 24YY) and in the across-environment BLUP dataset. (**A**) shows results for U1/HAE; (**B**) for U1/PH. Overlapping regions represent genes consistently supported by multiple environments.

To identify candidate genes underlying U1/HAE and U1/PH, we performed colocalization analysis using phenotypic data from both individual environments and their BLUPs. For U1/HAE, 23 genes were consistently identified between the 24YY environment and BLUPs, and three genes overlapped between 24GS and BLUPs (Figure 3A). For U1/PH, 17 genes overlapped in the 24YY-BLUP comparison, and two genes were shared between 24GS and BLUPs (Figure 3B). Based on peak SNPs from Manhattan plots, we further screened candidate genes within the stable QTL intervals *qU1*, *qU2*, *qU3*, and *qU4* (Table 2). No annotated genes were found in the *qU1* and *qU3* regions. Within the *qU2* interval (chr7: 148.32–148.51 Mb), ten candidate genes were identified, including the receptor-like protein kinase *Zm00001d021303* (putatively involved in cell signaling), endoglucanase 2 *Zm00001d021304* (associated with cell wall polysaccharide degradation), endoplasmic reticulum lumen protein-retention receptor family protein *Zm00001d021306*, and triosephosphate isomerase *Zm00001d021310* (implicated in glycolytic metabolism). The *qU4* interval (chr5: 6.71–6.81 Mb) contained thirteen candidate genes, such as gibberellin-regulated protein 10 *Zm00001d013222* (a growth-related regulator), the microtubule-associated protein *Zm00001d013224* (*MOR1*), the transcription factor *Zm00001d013232* (*bHLH113*), and arginine N-methyltransferase 2 *Zm00001d013219*, along with several genes of unknown function. These candidate genes are significantly associated with the proportional traits of the first internode and are implicated in diverse biological processes, including hormone response, cell wall dynamics, signal transduction, and metabolic regulation. Their identification provides a functional roadmap for further elucidation of the genetic networks controlling maize internode proportion and plant architecture.

### 3.4. Expression Profiling and Haplotype Analysis of Key Candidate Genes

**Figure 4:**
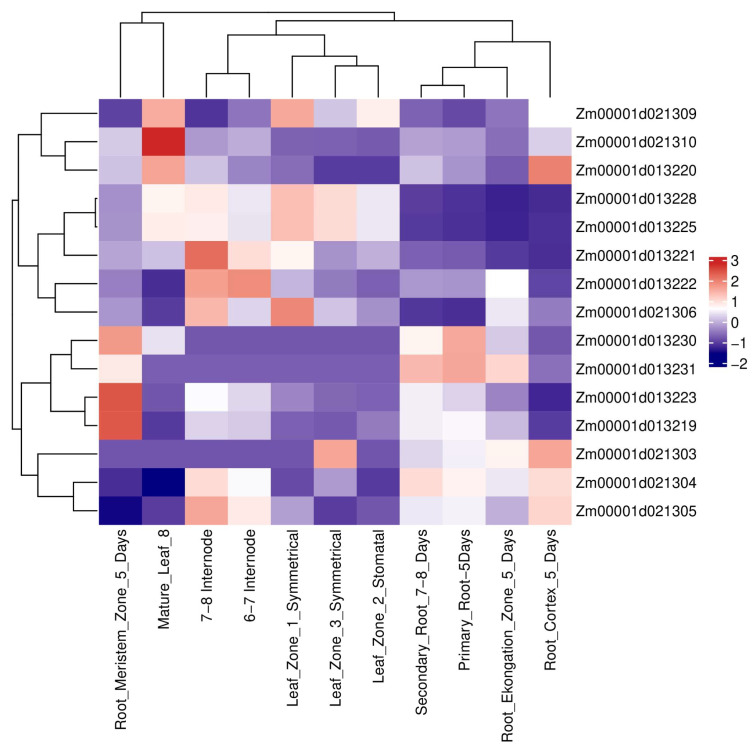
Expression profiles of key candidate genes across different maize tissues.

To evaluate the biological relevance of candidate genes underlying U1/HAE and U1/PH, we examined their expression patterns using publicly available transcriptome data (Figure 4). Among the 23 genes located in colocalized intervals, *Zm00001d021304* (encoding endoglucanase 2), *Zm00001d013221* (a domain-containing protein), *Zm00001d013222* (gibberellin-stimulated-like 10), and *Zm00001d013223* (retrovirus-related Pol polyprotein) displayed notably higher expression in roots, stems, and leaves relative to other candidates, supporting their potential roles in vegetative growth and development.

**Figure 5:**
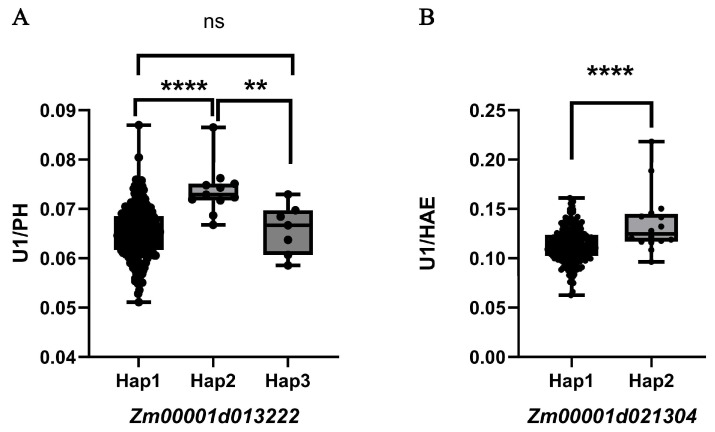
Haplotype analysis of (**A**) *Zm00001d013222* and (**B**) *Zm00001d021304*. Note: **** *p* < 0.0001; ** *p* < 0.01; ns, not significant.

Given that internode elongation is coordinately regulated by multiple hormones, we focused on two functionally annotated genes: *Zm00001d013222*, implicated in gibberellin signaling [23], and *Zm00001d021304*, involved in cell wall remodeling [24]. Using BLUP phenotypes from both environments, we performed haplotype-based association analysis for these genes (Figure 5; Table 3 and Table 4). For *Zm00001d013222*, three haplotypes were identified: Hap1 (ATACAC), Hap2 (GCGAGG), and Hap3 (ATACAG). Hap1 and Hap3 did not differ significantly in U1/PH, but both produced significantly lower U1/PH values than Hap2 (*p* < 0.01). This haplotype-dependent phenotypic variation supports *Zm00001d013222* as a candidate regulator of U1/PH. For *Zm00001d021304*, two haplotypes were found: Hap1 (GGTA) and Hap2 (TTAG). Hap2 exhibited a significantly higher U1/HAE value compared to Hap1 (*p* < 0.001), confirming *Zm00001d021304* as a strong candidate for U1/HAE. These results highlight *Zm00001d013222* and *Zm00001d021304* as promising genetic determinants of internode proportion, operating through hormone-mediated growth regulation and cell wall dynamics, respectively.

## 4. Discussion

Currently, with increasing demands for high-density planting in agriculture, maize production faces emerging challenges, such as greater lodging susceptibility, highlighting an urgent need for moderately dwarf hybrid maize varieties [25]. The maize “smart plant architecture” gene lac1, encoding a brassinosteroid C-22 hydroxylase, regulates upper leaf angle [26]. Key QTL controlling compact plant architecture in maize (*UPA1*/*brd1*, *UPA2*/*RAVL1*) have been successfully cloned, and the introgression of teosinte ligule alleles has been used to modify plant architecture. These improvements reduce resource competition under dense planting and significantly increase maize yield under high-density conditions [27]. In the present study, the ratios of U1 to HAE and to PH remained stable across two environments and BLUP values, and they were significantly higher than those of the other upper internodes. This suggests that modulating the U1 proportion could reduce redundant growth of the upper internodes without altering overall plant height. For example, a moderate reduction in the U1 ratio may shorten the first internode above the ear, lower the plant’s center of gravity, and thereby decrease stalk lodging risk. Previous studies have frequently identified the first internode above the ear as a critical site prone to breakage during stalk failure [14,15]. Moreover, the “U1/HAE” and “U1/PH” ratios represent more stable genetic traits than individual phenotypic measurements. In summary, by focusing on U1 ratio traits, this study not only enriches the genetic understanding of maize plant architecture regulation but also provides directly applicable resources for lodging-resistance breeding through the identification of stable QTL and candidate genes.

Maize internode elongation is coordinately regulated by multiple hormonal pathways. Transcription factors such as *ZmABI7* and *ZmMYB117* directly bind to the promoters of target genes (*ZmCYC1*, *ZmCYC3*, *ZmCYC7*, and *ZmCPP1*) to modulate cell cycle progression and cell wall modification, thereby influencing internode development [28]. Gibberellin (GA) shapes maize stem morphology by regulating peroxidase activity and thus cell wall lignification [29]. In rice, *ACE1* encodes a protein of unknown function that confers cell division capacity in the intercalary meristem, leading to GA-dependent internode elongation [30]. Another rice gene, *PINE1,* encodes a zinc-finger transcription factor that reduces stem sensitivity to GA; the floral meristem enhances stem responsiveness to GA by downregulating PINE1 expression [31]. These studies collectively underscore the pivotal role of plant hormones, particularly GA, in internode development. Consistently, among our colocalized candidate genes, *Zm00001d013222* encodes a gibberellin-regulated protein 10 (GSL10), which has been implicated in growth and developmental regulation [32]. Based on its functional annotation, we propose that *Zm00001d013222* is a key candidate gene regulating the U1/PH trait in this study, likely affecting stem growth and development by modulating gibberellin synthesis or metabolism in maize stems, ultimately leading to alterations in stem morphology.

During stem growth and development, the vascular cambium continuously proliferates, producing new cells. Xylem, which conducts water, differentiates inward from the cambium, while phloem—the main conduit for photoassimilate transport—differentiates outward. The completion of cell division relies on the precise assembly of a new cell wall between daughter cells [33]. Cellulose microfibrils are crucial for the structural organization of plant cell walls, allowing plants to sustain turgor-driven growth habits [34]. Endo-1,4-β-D-glucanases (EGases) are also speculated to function in cell expansion. Although their roles in cell wall synthesis and remodeling are not fully resolved, these enzymes cleave β-1,4-glycosidic bonds in polysaccharides, such as cellulose and xyloglucan [35]. Together, these reports highlight the central importance of the cell wall in stem growth, development, and morphogenesis. In our study, *Zm00001d021304* (*EG2*), a candidate gene colocalized with U1/HAE, encodes an endoglucanase belonging to glycoside hydrolase family 9 (GH9; also termed cellulase). It participates in multiple key processes of cell wall metabolism in higher plants, including the regulation of cellulose biosynthesis and degradation, modification of other (1,4)-β-glucan-containing wall polysaccharides, and mediation of cell wall loosening during cell elongation [24]. Analysis of *EG2* expression profiles using the qTeller MaizeGDB platform (https://qteller.maizegdb.org) revealed that its expression peaked 24 days before pollination and remained relatively stable during other periods, suggesting involvement in the stem-jointing stage of maize. *EG2* may promote the degradation of cell wall polysaccharides to maintain wall plasticity, thereby facilitating stem elongation. Notably, the previously reported *stiff1* gene enhances stalk strength and lodging resistance by negatively regulating cellulose and lignin synthesis and influencing cell wall thickness [36]. This gene may synergize with the identified *EG2* gene to balance internode proportion optimization and stem strength enhancement dynamically, a core objective of lodging-resistant plant architecture breeding.

## 5. Conclusions

Through a GWAS approach in a diverse maize panel, we identified stable QTL (*qU1*–*qU4*) and candidate genes governing the proportion of the first internode above the ear. Two functionally annotated genes, *Zm00001d013222* (involved in gibberellin signaling) and *Zm00001d021304* (a cell wall-modifying endoglucanase), exhibited significant haplotype-based associations with U1/PH and U1/HAE, respectively. These findings highlight the coordinated roles of hormone pathways and cell wall metabolism in shaping maize internode architecture. The stability of these loci across environments underscores their potential value for molecular breeding. This study provides novel genetic insights and precise targets for improving plant architecture and lodging resistance, supporting the development of high-yield maize varieties optimized for modern high-density cropping systems.

## Figures and Tables

**Table 1:** Descriptive statistics for internode-related traits of the maize association panel under two environments and their best linear unbiased predictions (BLUPs).

Trait	Environment	Range	Mean	SD	Ske.	Kur.	CV (%)	Heritability
PH (cm)	24GS	139.33–290.33	210.42	27.75	0.1	0.57	0.13	0.71
	24YY	143.00–300.00	207.17	29.44	0.47	−0.18	0.14	
	BLUP	169.68–257.70	208.51	16.34	0.37	0.07	0.08	
EH (cm)	24GS	30.00–132.00	83.36	18.9	0.15	−0.32	0.23	0.69
	24YY	47.33–161.67	90.08	21.23	0.54	0.12	0.24	
	BLUP	55.21–122.72	86.89	10.9	0.43	0.10	0.13	
HAE (cm)	24GS	70.67–186.33	127.06	19.3	−0.08	0.37	0.15	0.63
	24YY	77.67–178.33	117.10	19.61	0.22	−0.28	0.17	
	BLUP	92.33–156.06	121.62	10.95	0.12	−0.15	0.09	
Zero (cm)	24GS	6.00–19.67	13.25	2.45	0.06	0.17	0.18	0.32
	24YY	6.50–21.50	13.01	2.39	0.25	0.63	0.18	
	BLUP	11.33–14.66	13.10	0.47	0.14	0.79	0.04	
U1 (cm)	24GS	8.00–18.67	14.07	1.81	0.09	0.44	0.13	0.49
	24YY	6.67–21.67	13.32	2.43	0.23	0.40	0.18	
	BLUP	11.56–16.14	13.61	0.75	0.32	0.66	0.06	
U2 (cm)	24GS	6.00–22.00	12.99	2.40	0.35	1.24	0.18	0.35
	24YY	5.00–25.00	12.52	2.49	0.29	2.16	0.20	
	BLUP	10.74–15.13	12.64	0.68	0.28	0.95	0.05	
U3 (cm)	24GS	6.00–19.33	12.91	2.37	−0.20	0.24	0.18	0.39
	24YY	5.50–19.67	11.95	2.38	0.03	0.15	0.20	
	BLUP	10.14–14.38	12.38	0.67	−0.01	0.38	0.05	
U4 (cm)	24GS	4.50–19.33	12.67	2.53	−0.17	0.67	0.20	0.47
	24YY	3.00–27.50	11.67	2.78	0.45	3.72	0.24	
	BLUP	6.92–17.75	12.14	1.26	0.03	2.03	0.10	
U5 (cm)	24GS	5.00–20.33	12.49	2.70	−0.17	0.68	0.22	0.58
	24YY	4.00–22.00	11.59	2.78	0.22	1.10	0.24	
	BLUP	8.41–16.73	12.01	1.18	0.30	1.59	0.10	
U_AVE (cm)	24GS	6.80–18.73	13.03	1.94	−0.13	0.68	0.15	0.55
	24YY	6.46–20.63	12.18	2.07	0.20	0.85	0.17	
	BLUP	10.41–15.28	12.56	0.73	0.31	1.36	0.06	
U1/HAE	24GS	0.07–0.17	0.11	0.01	0.72	0.71	0.15	0.59
	24YY	0.06–0.22	0.12	0.02	1.05	3.83	0.17	
	BLUP	0.09–0.15	0.11	0.01	0.56	0.94	0.08	
U2/HAE	24GS	0.07–0.20	0.10	0.02	1.35	4.52	0.18	0.20
	24YY	0.05–0.24	0.11	0.02	1.48	8.02	0.18	
	BLUP	0.09–0.13	0.10	0.01	0.44	0.63	0.07	
U3/HAE	24GS	0.06–0.18	0.10	0.02	0.78	2.91	0.16	0.39
	24YY	0.06–0.14	0.10	0.02	0.21	−0.18	0.15	
	BLUP	0.09–0.13	0.10	0.01	0.54	0.99	0.07	
U4/HAE	24GS	0.04–0.19	0.10	0.02	0.96	4.42	0.18	0.50
	24YY	0.04–0.19	0.10	0.02	0.52	2.60	0.19	
	BLUP	0.07–0.14	0.10	0.01	0.56	2.32	0.09	
U5/HAE	24GS	0.05–0.20	0.10	0.02	0.98	4.49	0.20	0.51
	24YY	0.05–0.15	0.10	0.02	0.05	0.74	0.18	
	BLUP	0.08–0.14	0.10	0.01	0.75	2.13	0.08	
U_AVE/HAE	24GS	0.08–0.18	0.10	0.01	1.45	6.57	0.13	0.60
	24YY	0.07–0.15	0.10	0.01	0.56	0.69	0.12	
	BLUP	0.09–0.14	0.10	0.01	0.72	2.10	0.06	
U1/PH	24GS	0.04–0.10	0.07	0.01	0.47	0.32	0.16	0.65
	24YY	0.03–0.13	0.06	0.01	0.65	2.77	0.19	
	BLUP	0.05–0.09	0.07	0.01	0.31	1.05	0.08	
B1 (cm)	24GS	8.33–23.00	13.80	2.22	0.62	1.62	0.16	0.06
	24YY	5.00–26.33	14.72	3.39	0.56	1.08	0.23	
	BLUP	13.52–16.42	14.34	0.28	1.61	9.65	0.02	
B2 (cm)	24GS	6.67–19.33	13.44	2.39	−0.05	−0.36	0.18	0.43
	24YY	7.33–20.67	14.00	2.30	0.09	−0.11	0.16	
	BLUP	11.85–15.72	13.71	0.72	0.26	−0.11	0.05	
B3 (cm)	24GS	4.00–19.33	12.19	2.71	0.01	−0.17	0.22	0.53
	24YY	5.50–23.33	13.61	2.72	0.12	0.31	0.20	
	BLUP	9.26–16.32	12.92	0.10	0.22	0.59	0.08	
B4 (cm)	24GS	5.33–17.00	11.01	2.67	0.11	−0.77	0.24	0.52
	24YY	6.33–21.00	12.67	2.64	0.40	0.44	0.21	
	BLUP	9.55–14.24	11.88	0.91	0.26	−0.06	0.08	
B5 (cm)	24GS	4.00–16.00	10.31	2.61	0.14	−0.31	0.25	0.41
	24YY	5.00–19.67	11.28	2.77	0.28	−0.38	0.25	
	BLUP	9.59–12.30	10.82	0.51	0.35	−0.25	0.05	
B1/EH	24GS	0.09–0.32	0.17	0.04	0.48	0.70	0.22	0.52
	24YY	0.06–0.35	0.17	0.05	0.67	0.95	0.28	
	BLUP	0.12–0.25	0.17	0.02	0.25	0.41	0.12	
B2/EH	24GS	0.10–0.28	0.17	0.03	0.54	1.52	0.17	0.61
	24YY	0.08–0.30	0.16	0.04	0.55	0.79	0.24	
	BLUP	0.11–0.22	0.16	0.02	0.03	0.44	0.11	
B3/EH	24GS	0.08–0.20	0.15	0.02	0.03	0.57	0.12	0.50
	24YY	0.07–0.32	0.16	0.03	0.64	2.89	0.20	
	BLUP	0.11–0.21	0.15	0.01	−0.08	0.70	0.09	
B4/EH	24GS	0.09–0.22	0.13	0.02	1.10	4.54	0.14	0.29
	24YY	0.09–0.31	0.14	0.02	1.56	8.23	0.17	
	BLUP	0.10–0.18	0.14	0.01	0.25	1.61	0.08	
B5/EH	24GS	0.06–0.15	0.11	0.02	−0.28	0.16	0.16	0.12
	24YY	0.08–0.18	0.13	0.02	0.34	0.28	0.13	
	BLUP	0.10–0.18	0.12	0.01	0.46	1.21	0.09	
Zero/HAE	24GS	0.06–0.17	0.10	0.02	0.56	1.50	0.15	0.26
	24YY	0.07–0.22	0.11	0.02	1.29	3.98	0.18	
	BLUP	0.09–0.14	0.11	0.01	0.40	0.11	0.08	

Notes: U1–U5, the five internodes above the ear (numbered from the lowest to the highest); B1–B5, the five internodes below the ear (numbered from the highest to the lowest); Zero, the ear-position internode; U_AVE, the mean length of the five internodes above the ear. SD, standard deviation; Ske., skewness; Kur., kurtosis; CV, coefficient of variation.

**Table 2:** Significant SNPs and candidate genes associated with internode proportion traits in maize.

Trait	QTL Name	Environment	Peak SNP	Chr	Position (bp) a	*p* Valued b	R^2^ (%) c	Candidate Gene	Function
U1/HAE	*qU1*	BLUP&24YY	chr1.s_165467245	1	165,467,245	3.41 × 10^−7^	9.79		
U1/HAE	*qU2*	BLUP&24YY	chr7.s_148457037	7	148,457,037	3.24 × 10^−7^	9.55	*Zm00001d021302*	Unknown function
								*Zm00001d021303*	Probable receptor-like protein kinase
								*Zm00001d021304*	Endoglucanase 2
								*Zm00001d021305*	Seipin-2
								*Zm00001d021306*	ER lumen protein retaining receptor family protein
								*Zm00001d021309*	Transcription initiation factor IIB-2
								*Zm00001d021310*	Triosephosphate isomerase
								*Zm00001d021311*	AP-3 complex subunit mu
								*Zm00001d021312*	Unknown function
								*Zm00001d001244*	Unknown function
U1/PH	*qU3*	BLUP&24YY	chr1.s_165433756	1	165,433,756	3.53 × 10^−6^	10.55		
U1/PH	*qU4*	BLUP&24YY	chr5.s_6757775	5	6,757,775	1.27 × 10^−6^	9.73	*Zm00001d013219*	Arginine N-methyltransferase 2
								*Zm00001d013220*	Probable protein phosphatase 2C 48
								*Zm00001d013221*	SWIb domain-containing protein
								*Zm00001d013222*	Gibberellin-regulated protein 10
								*Zm00001d013223*	Retrovirus-related Pol polyprotein LINE-1
								*Zm00001d013224*	Protein MOR1
								*Zm00001d013225*	TIP41-like family protein
								*Zm00001d013226*	Cytochrome P450 family 81 subfamily D polypeptide 8
								*Zm00001d013228*	TIP41-like family protein
								*Zm00001d013229*	Cytochrome P450 CYP81A17
								*Zm00001d013230*	Bentazon resistance1; recessive susceptibility to nicosulfuron (Accent) and mesotrione herbicides
								*Zm00001d013231*	Cytochrome P450 family 81 subfamily D polypeptide 8
								*Zm00001d013232*	Transcription factor bHLH113

Notes: ^a^ Physical position in base pairs for the peak SNP according to B73_RefGen_v4 of the maize reference sequence. Physical interval for each QTL was assigned as 100 kb according to LD decay (50 kb upstream and downstream of the peak SNP, which is the SNP with the lowest *p*-value); ^b^ *p*-value of the corresponding trait calculated by the Q + K model; ^c^ the phenotypic variance explained by the corresponding locus.

**Table 3:** Haplotype analysis of *Zm00001d013222*.

	SNP6757087	SNP6757775	SNP6757793	SNP6757796	SNP6757862	SNP6774451
Hap1	A	T	A	C	A	C
Hap2	G	C	G	A	G	G
Hap3	A	T	A	C	A	G

**Table 4:** Haplotype analysis of *Zm00001d021304*.

	SNP148365698	SNP148365753	SNP148365570	SNP148365390
Hap1	G	G	T	A
Hap2	T	T	A	G

## Data Availability

The raw data supporting the conclusions of this article will be made available by the authors on request.

## Supplementary Materials

The following supporting information can be downloaded at https://www.mdpi.com/article/10.3390/genes17020141/s1. Figure S1. Schematic diagram of the morphological structure of the maize plant. Figure S2. Phenotypic distribution of internode-related traits across two environments and their best linear unbiased predictions (BLUPs). Figure S3. Distribution and correlation of PH, EH, and IL above the ear. Figure S4. Proportions of individual IL above the ear relative to HAE and PH across different environments (A,D) 24GS; (B,E) 24YY; (C,F) BLUP. Figure S5 Circular Manhattan plots of (A) B1, (B) B2, (C) B3, (D) B4, (E) B5, (F) U1, (G) U2, (H) U3, (I) U4, (J) U5, (K) U_AVE, (L) Zero, (M) PH, (N) EH and (O) HAE. For each Manhattan plot, from inner to outer rings represent 24GS, 24YY and BLUP, respectively. Figure S6 Circular Manhattan plots of (A) B1/EH, (B) B2/EH, (C) B3/EH, (D) B4/EH, (E) B5/EH, (F) U1/HAE, (G) U2/HAE, (H) U3/HAE, (I) U4/HAE, (J) U5/HAE, (K) U_AVE/HAE, (L) Zero/HAE and (M) U1/PH. For each Manhattan plot, from inner to outer rings represent 24GS, 24YY and BLUP, respectively. Figure S7 Q-Q plots of (A) B1, (B) B2, (C) B3, (D) B4, (E) B5, (F) U1, (G) U2, (H) U3, (I) U4, (J) U5, (K) U_AVE, (L) Zero, (M) PH, (N) EH and (O) HAE. Figure S8 Q-Q plots of (A) B1/EH, (B) B2/EH, (C) B3/EH, (D) B4/EH, (E) B5/EH, (F) U1/HAE, (G) U2/HAE, (H) U3/HAE, (I) U4/HAE, (J) U5/HAE,(K) U_AVE/HAE, (L) Zero/HAE and (M) U1/PH. Table S1. All genes within significant QTL associated with IL-related traits by GWAS. Table S2. All genes within the QTL intervals significantly associated with internode length (IL)-related traits that were co-localized across different environments and identified by GWAS.
