# Supplementary material for: Genetic Mapping Identifies Stable QTL and Candidate Genes Regulating Internode Proportion for Maize Plant Architecture Improvement
Source: Genes (Basel). 2026 Jan 27;17(2):141. doi: 10.3390/genes17020141 (PMC12940924; doi:10.3390/genes17020141)
Supplement: Supplementary file 1 [file genes-17-00141-s001.zip › Figure S1-8.pdf]

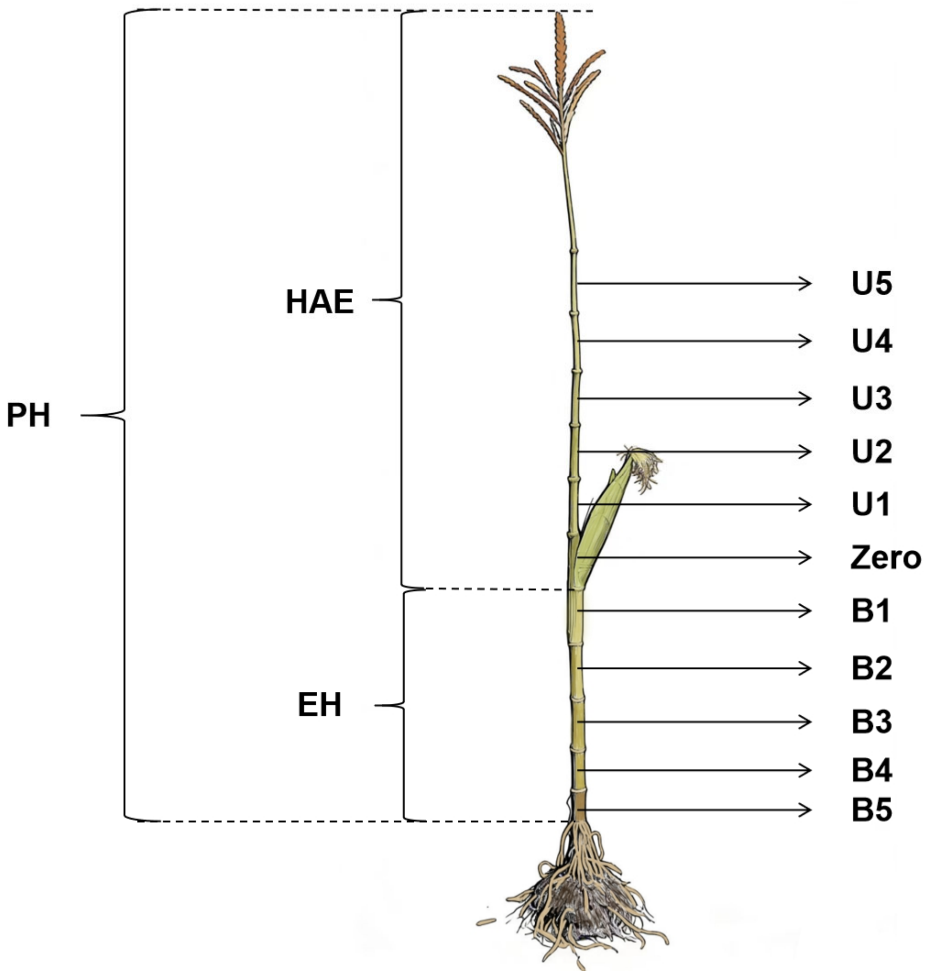

Figure S1. Schematic diagram of the morphological structure of the maize plant.

Note: Plant height (PH), ear height (EH), height above ear (HAE), lengths of internodes above the ear (U1–U5), lengths of internodes below the ear (B1–B5), the ear-position internode (Zero)

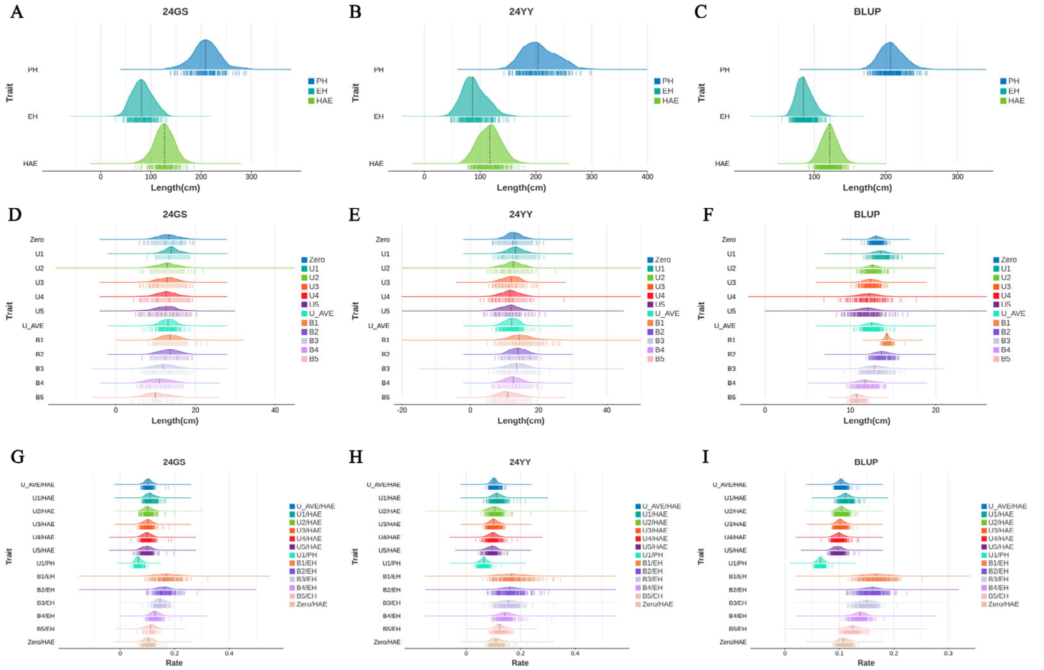

Figure S2. Phenotypic distribution of internode-related traits across two environments and their best linear unbiased predictions (BLUPs).

Notes: A-C, show the frequency distributions of PH, EH, and HAE in the 24GS, 24YY, and BLUP datasets, respectively. D-F, present the corresponding distributions of internode lengths in maize. G-I, present the frequency distributions of the ratios of maize internode lengths to PH, EH, and HAE. Vertical lines indicate the phenotypic values of each trait for every inbred line.

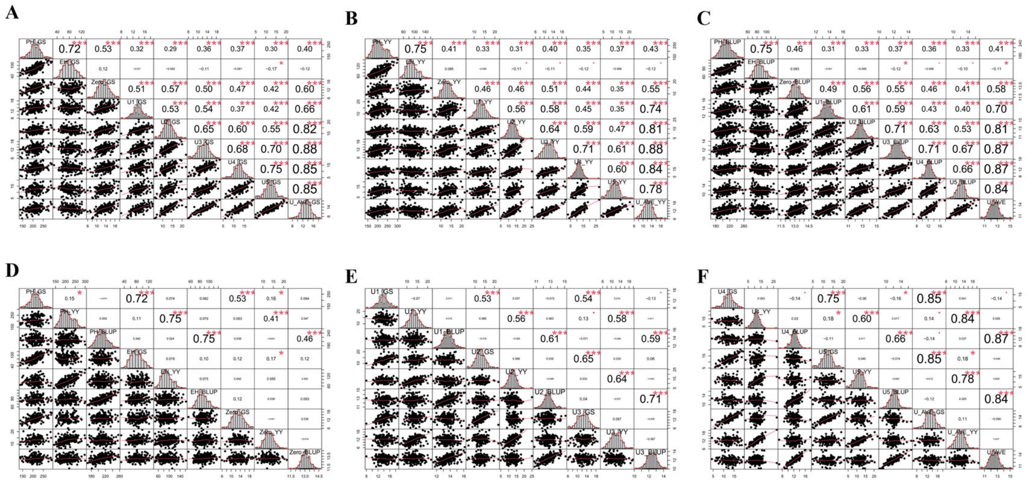

Figure S3. Distribution and correlation of PH, EH, and IL above the ear.

Notes: (A, B, C) Correlation of PH, EH, and IL above the ear within the same environment. (D,E,F) Correlation of the same traits between different environments. Frequency distribution histograms of traits are located on the diagonal; the area below the diagonal shows scatter plots of traits, and the area above presents the correlation coefficients between each pair of traits. * indicates significance at $p < 0.05$. ** indicates significance at $p < 0.01$. *** indicates significance at $p < 0.001$.

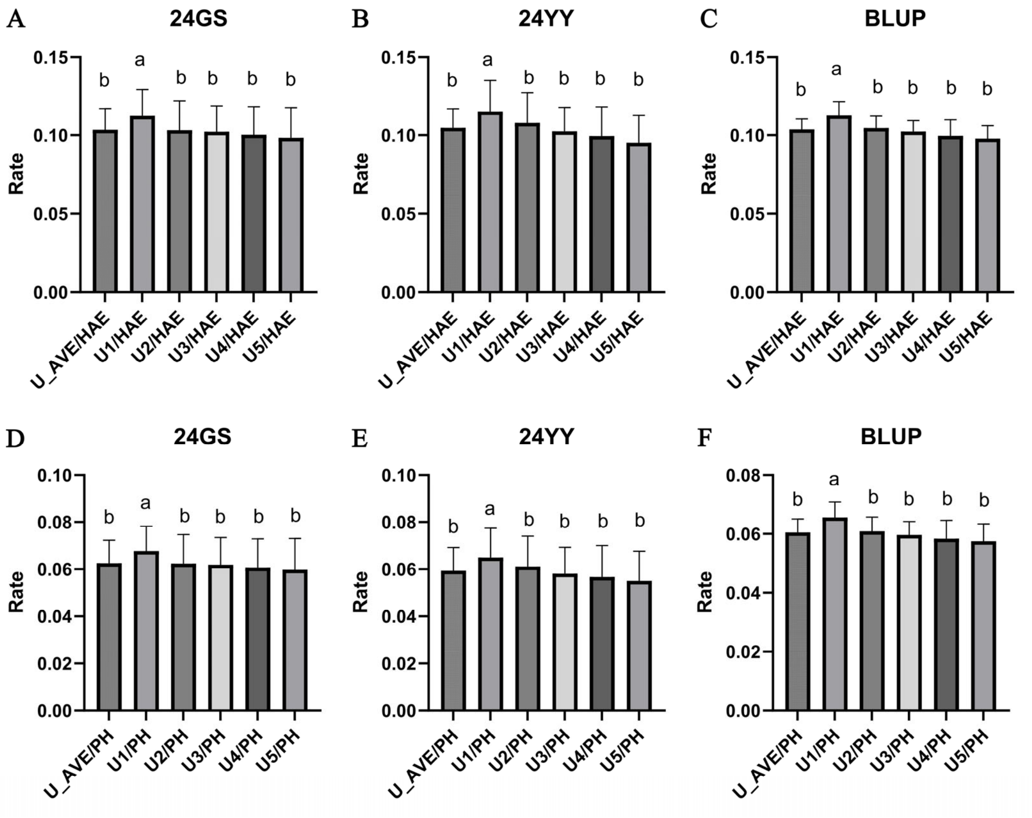

Figure S4. Proportions of individual IL above the ear relative to HAE and PH across different environments (A,D) 24GS; (B,E) 24YY; (C,F) BLUP.

Notes: Letters a and b indicate extremely significant differences at $p < 0.0001$.

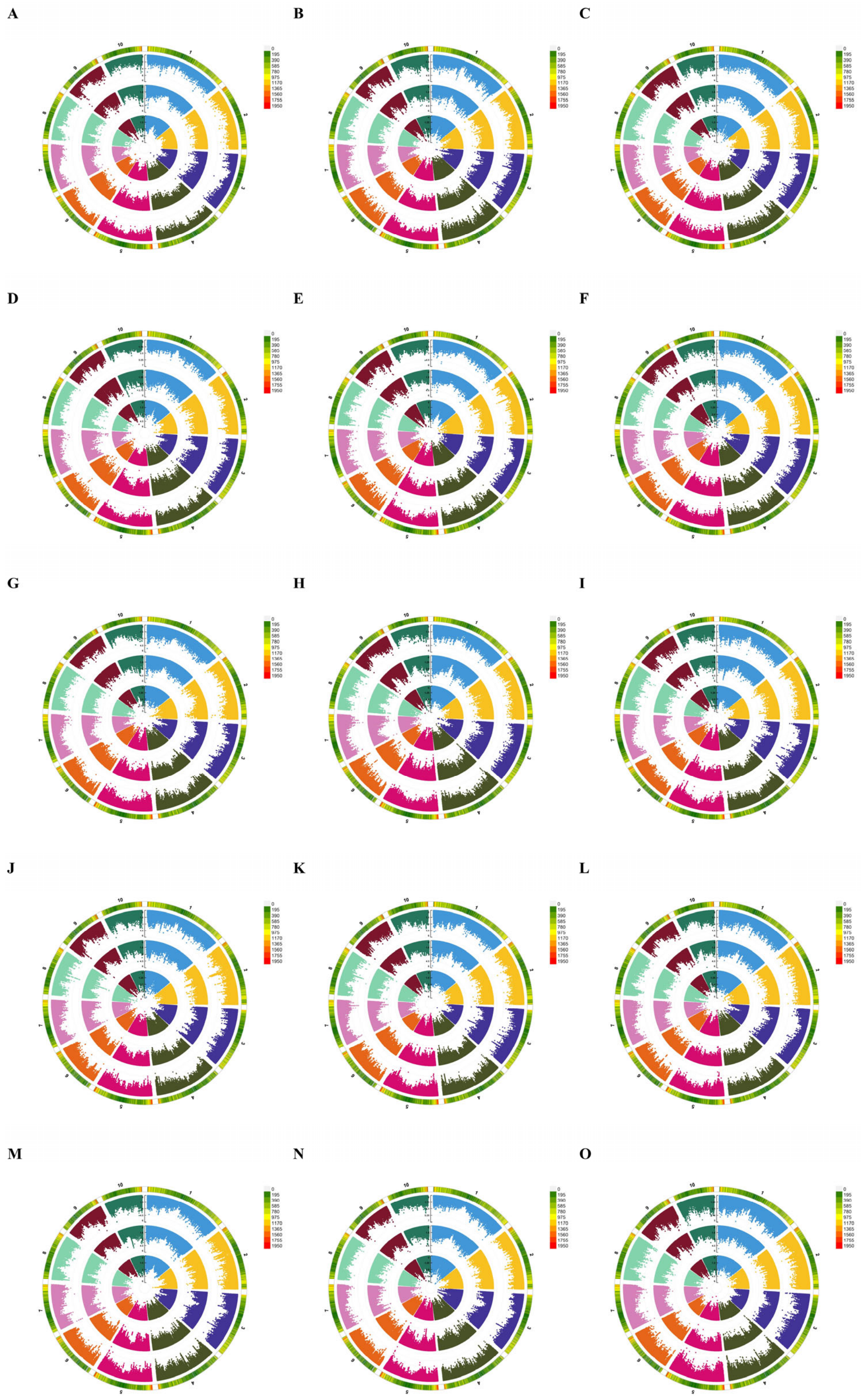

Figure S5 Circular Manhattan plots of (A) B1, (B) B2, (C) B3, (D) B4, (E) B5, (F) U1, (G) U2, (H) U3, (I) U4, (J) U5,(K) U_AVE,(L)Zero,(M)PH,(N) EH and (O) HAE. For each Manhattan plot, from inner to outer rings represent 24GS, 24YY and BLUP, respectively

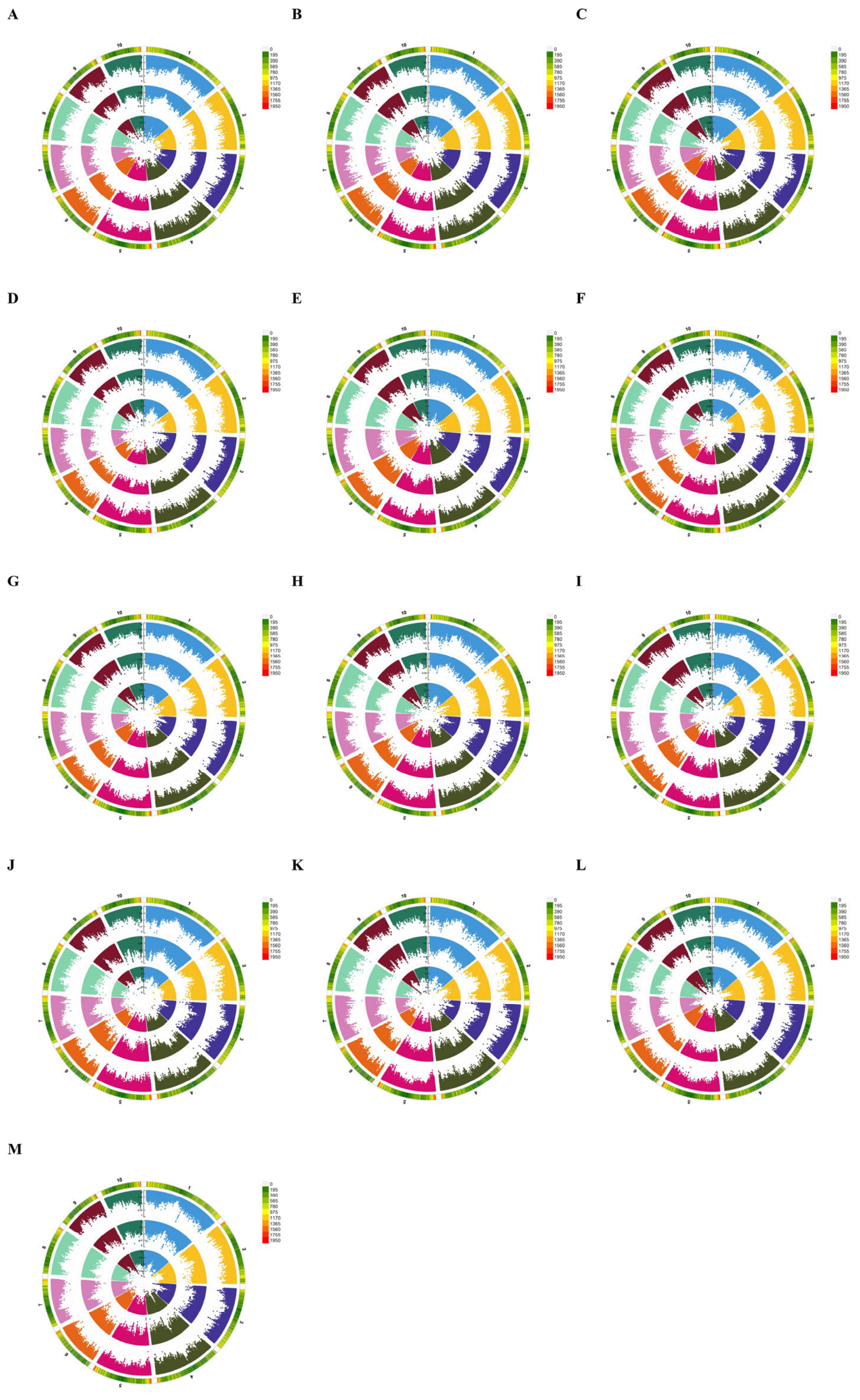

Figure S6 Circular Manhattan plots of (A) B1/EH, (B) B2/EH, (C) B3/EH, (D) B4/EH, (E) B5/EH, (F) U1/HAE, (G) U2/HAE, (H) U3/HAE, (I) U4/HAE, (J) U5/HAE,(K) U_AVE/HAE ,(L)Zero/HAE and (M)U1/PH. For each Manhattan plot, from inner to outer rings represent 24GS, 24YY and BLUP, respectively.

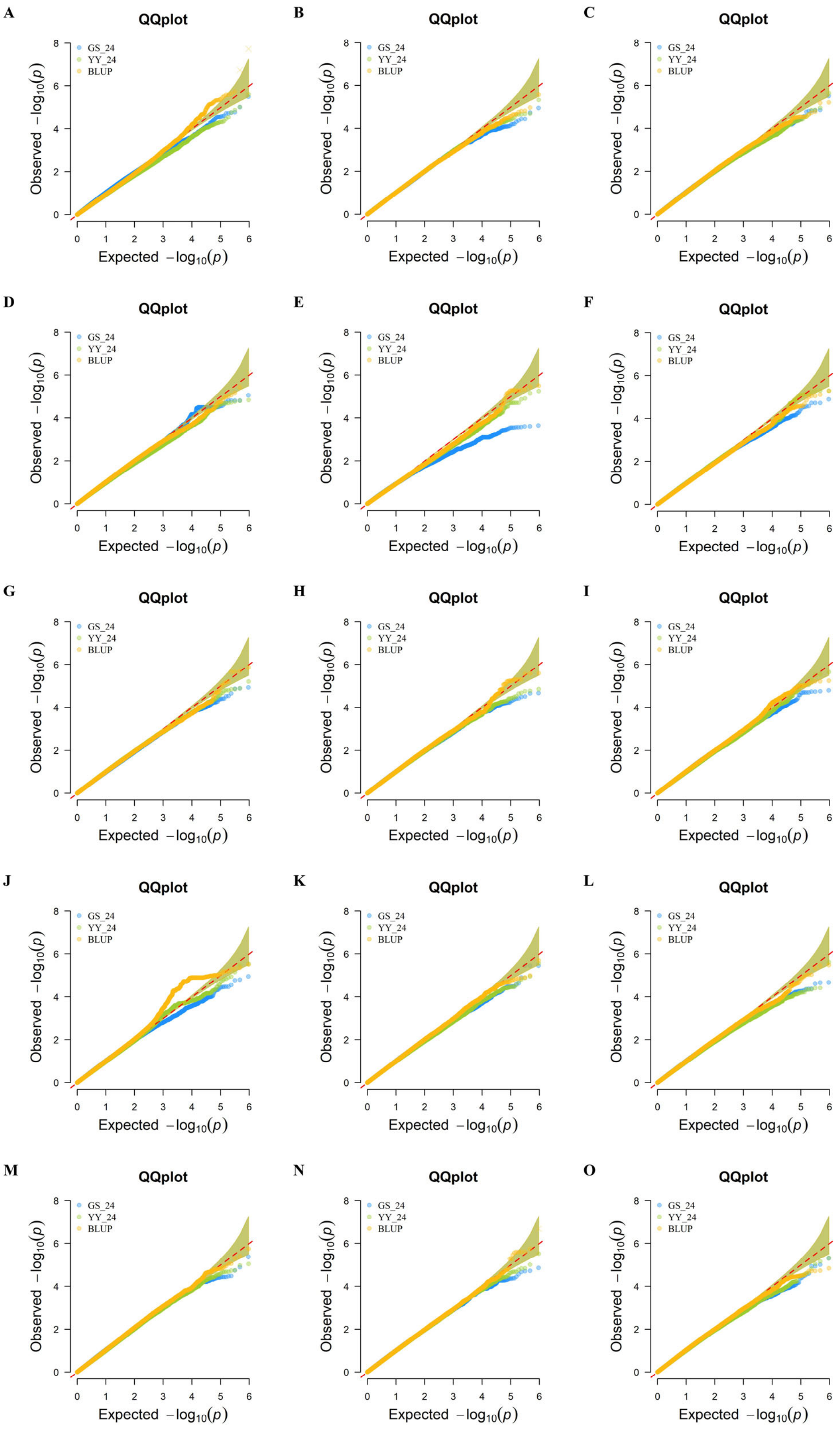

Figure S7 Q-Q plots of (A) B1, (B) B2, (C) B3, (D) B4, (E) B5, (F) U1, (G) U2, (H) U3, (I) U4, (J) U5,(K)U_AVE,(L)Zero,(M)PH,(N)EH and (O) HAE.

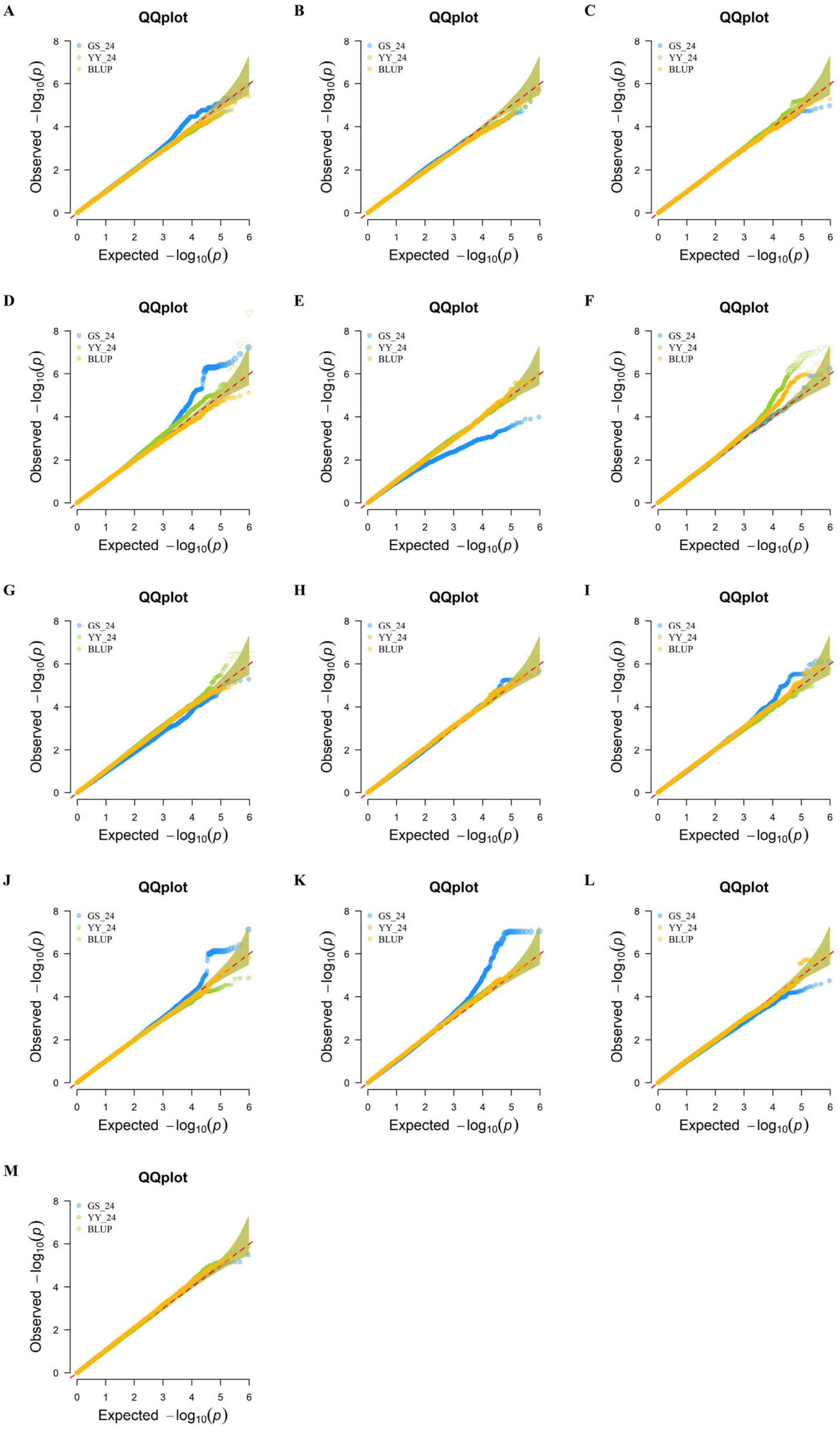

Figure S8 Q-Q plots of (A) B1/EH, (B) B2/EH, (C) B3/EH, (D) B4/EH, (E) B5/EH, (F) U1/HAE, (G) U2/HAE, (H) U3/HAE, (I) U4/HAE, (J) U5/HAE,(K) U_AVE/HAE ,(L)Zero/HAE and(M)U1/PH.
